# Antioxidant Activity of *Urtica dioica*: An Important Property Contributing to Multiple Biological Activities

**DOI:** 10.3390/antiox11122494

**Published:** 2022-12-19

**Authors:** Varun Jaiswal, Hae-Jeung Lee

**Affiliations:** 1 Department of Food and Nutrition, College of BioNano Technology, Gachon University, 1342 Seongnam-daero, Sujeong-gu, Seongnam-si 13120, Republic of Korea; 2 Institute for Aging and Clinical Nutrition Research, Gachon University, Seongnam-si 13120, Republic of Korea; 3 Department of Health Sciences and Technology, GAIHST, Gachon University, Incheon 21999, Republic of Korea

**Keywords:** antioxidant, organs, diabetes, cancer, functional food, medicinal plant, clinical trials, in vivo, *Urtica dioica*

## Abstract

*Urtica dioica* (UD) is a multi-functional plant known to be used as both food and medicine from ancient times. The plant has the potential to be used as a fertilizer and for biological pest control. It is also used in textile and related industries for its quality fibers. In the recent past, the plant has received great attention for its numerous important biological activities and food applications. The antioxidant activity of UD is the crucial factor supporting its important biological activities, such as anticancer, antidiabetic and anti-inflammatory properties. The antioxidant activity of UD is also found to be protective in different organs, including the brain, liver, lungs, kidney, ovary, and uterus, and may also be protective against diseases associated with these organs. Few clinical studies have endorsed the antioxidant potential of UD in patients. The current work is an attempt to comprehensively compile and discuss the antioxidant activity of UD from in vitro, in vivo and human studies. The insights of the current study would be helpful in getting a panoramic view of the antioxidant potential of UD, and provide direction for optimizing and developing it for therapeutic applications against important diseases and conditions in the near future.

## 1. Introduction

Functional foods and supplements are gaining importance in recent times. Some medicinal plants which are edible can also serve as food along with their medicinal properties. These plants have the added advantage of being developed as functional foods, supplements and/or therapeutics for long term consumption.

*Urtica dioica* (UD), is a multi-functional plant commonly known as stinging nettle or common nettle, which has been used as wild vegetable for centuries [1,2,3]. UD is also considered an ancient medicinal plant that was used for arthritis and lumbago [1]. The name of the species *dioica* is adopted from the dioecious nature of plants in some subspecies. The genus name *Urtica* is of Latin origin and is believed to be derived from *uro* and *urere* meaning to burn and to sting, respectively. These properties of UD could be the reason for the initial medicinal usage of the herb against arthritis and paralysis, for increased blood circulation and bringing warmth to the affected area of the body [1]. 

UD is believed to be native to Eurasia and is currently distributed over almost all of the world. It is mostly found in North Africa, North America, Europe, and different regions in Asia [1]. UD is considered a weed in intensive agriculture due to its rapid growth, high spread, and soil coverage [4]. However, there are commercial and ecological reasons to cultivate UD, as it has multiple potential uses and supports flora and fauna. The members of the genus *Urtica* are herbaceous perennials, of them UD is most important.

The herb is mainly characterized by unicellular stinging hairs. It has long soft leaves with a sharp saw-toothed margin with an acuminate tip. The plant has a strong stem that is mostly unbranched and quadrangular. The leaves and stems are both hairy and contain many stinging hairs or trichomes that possess different chemicals (such as histamine, 5-hydroxytryptamine, and acetylcholine). These chemicals can be injected after dislodging of the tip and cause itching to the skin [5]. 

UD has an extraordinary nutritional profile; leaves of UD are rich in chlorophyll, carotenoid, proteins, vitamins, minerals, and bioactive compounds, such as flavonoids and phenolic acids [6,7]. These leaves are consumed as salads, soups, tea and vegetables in different countries; however, they are more commonly used in poorer countries. Recently, UD has received more attention as a highly nutritious food due to its increased use in different food products and its health-promoting properties [8]. 

The antioxidant properties of UD and its extract can be utilized in the food industry. Incorporation of extract and leaves of UD as functional ingredients can increase the shelf-life, quality and color of different foods. A water extract from UD was found to decrease the lipid peroxidation and increase the quality of meat balls [9] and sausage [10]. It was also found to increase the self-life of vacuum packaged beef streaks [11], ground beef [12] and minced meat of common Kilka (*Clupeonella cultriventris*) [13]. 

Although UD is mostly known for its medicinal and food applications, it was also used in the textile industry due to the presence of fiber. It was used to make textiles during the First World War in Austria and Germany [2]. A recent study supports the use of fiber from the upper stalk of UD for the textile industry, and the lower part for fibers in industrial applications (technical fibers) [2]. UD can also be used for pest control in the green house as it can source natural enemies of pests to protect greenhouse cultures [14]. It has shown the potential to be used as an organic fertilizer that can replace other conventional fertilizers [15,16].

Researchers have explored different medicinal properties of UD for a long time. Almost all parts of the plant, such as leaves, roots, flowers, seeds, and aerial parts, have been used for pharmacological activities [17]. These medicinal properties were studied against a number of diseases and conditions such as arteriosclerosis, rheumatism and sciatica, allergy, asthma, coughs, dandruff, diabetes, diarrhea, eczema, fever, gout, hemorrhoids, kidney stone, urinary tract infection, nose bleeds, scurvy, tuberculosis, obesity, cardiovascular diseases, neurodegenerative diseases and different cancers including lung, prostate, breast and cervical cancer [3,18,19,20,21]. The antioxidant activity of UD is believed to support most of these biological activities [18,21,22,23,24]. Oxidants such as reactive oxygen species (ROS) and reactive nitrogen species (RNS) are continuously produced in living systems, and these have a very short half-life. In living systems, these oxidants can cause damage to macromolecules such as DNA, proteins and lipids that may lead to disease conditions such as cancer, diabetes, cirrhosis, arteriosclerosis, asthma, cardiovascular diseases, neurodegenerative diseases, and arthritis [25,26]. Antioxidants can neutralize these reactive species through enzymatic and non-enzymatic antioxidant activities. Therefore, antioxidants that are used against these diseases were found to be effective [25,26].

Although synthetic antioxidants show higher stability, low cost, and higher availability, natural antioxidants are preferred over synthetic antioxidants. Long term or high consumption of synthetic antioxidants can cause serious side effects in the reproductive system, skin allergies, gastrointestinal tract problems and may increase the risk of some cancer [27,28,29,30,31]. Thus, the use of natural antioxidants is always sought and in this scenario, UD can be used as an important source of natural antioxidants. UD leaf powder was found to have a higher concentration of bioactive compounds, antioxidants, and antioxidant activity compared to wheat and barley flour [32]. UD leaf powder has also been utilized in the bakery industry to increase the nutritional value and antioxidant properties of bread [8].

Considering the huge medicinal potential of UD, several reviews have attempted to catalog the phytochemicals and different pharmacological properties of UD [3,18,33,34,35]. These reviews compiled the phytochemical, therapeutics, and all pharmacological properties of UD [35], or focused on some disease such as cancer, diabetes and cardiovascular diseases [18,19] reported in the literature. Some reviews also discussed the nutritional and other benefits of UD [33,35]. The antioxidant properties of UD were observed in a huge number of studies against various diseases. In some studies, the antioxidant activity of UD extract was found to be better, or comparable to, the reference compounds (such as butylated hydroxyanisole (BHA), butylated hydroxytoluene (BHT), and α-Tocopherol) [36]. However, the antioxidant properties of UD, which seems to be crucial for most of its important medicinal activities, are not systematically compiled and discussed for further development. The current work is an attempt to compile and discuss antioxidant activity from in vitro, in vivo and human studies. Insights from the current study will be helpful to get a panoramic view of the antioxidant potential of UD and provide direction to develop it for therapeutic applications against important diseases and conditions in the near future.

## 2. Electronic Literature Search

A literature search was carried out using online databases, such as PubMed, Google Scholar, Google, Scopus, and ResearchGate. Important keywords such as *Urtica dioica*, stinging nettle, common nettle, and their combinations with antioxidant, human studies, in vivo, in vitro, and clinical trials were used for the literature search. The resulting research articles, review articles, books, and theses published in the English language until 30 November 2022 were explored, and the important literature was included in the current study. Notably, research providing the antioxidant activities of UD mixed with other substances and metal nanoparticles were not included in the current study.

## 3. Antioxidant Potential of UD

**Figure 1:**
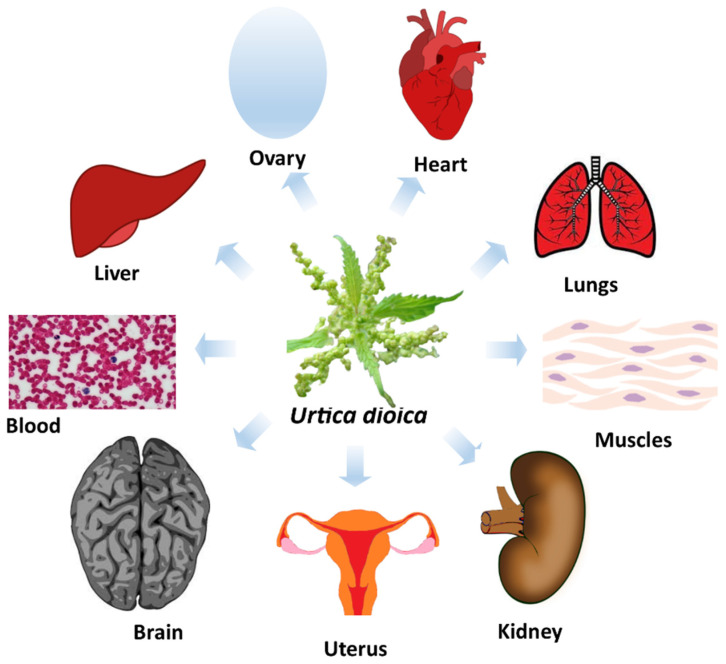
Antioxidant activity of UD observed in different organs.

The antioxidant activity of different parts of UD has been studied in several studies, including in vitro, in vivo, and human studies. Experiments have also been conducted to enhance antioxidant activities, by increasing the content of active phytochemicals in extracts of UD through optimization of extraction parameters and techniques. Effective antioxidant activity was observed in different standard in vitro and chemical based assays (Table 1). The beneficial effect of the antioxidant activity of UD in different organs was also observed in several in vivo studies (Figure 1). The effective antioxidant activity of UD was also detected in different organs in animal models (Figure 1), which also highlights the medicinal potential of UD against different diseases (Table 2). Human studies also support the antioxidant activities of UD leaves and root extract against different diseases in clinical trials (Table 3). The long history of UD as a vegetable food, supports the safety of long-term food supplement or therapeutic usage of UD. The antioxidant activity of extract from different part of UD, studied through in vitro methods, is described in Table 1. Notably, the experiments in which the value of antioxidant activity was not provided (results of antioxidant activities are only provided in graph/images) are not included in the table [37,38,39].

### 3.1. In Vitro Antioxidant Studies

In an initial study to analyze the antioxidant activity of twenty-seven non-cultivated vegetables in southern Italy, UD was studied. Free radical scavenging activity (FRSA) was analyzed in the 1,1-diphenyl-2-picrylhydrazil radical (DPPH) assay. Inhibition of lipid peroxidation in liposomes and inhibition of xanthine oxidase were analyzed for all selected plants. The antioxidant activity of the UD leaf extract for inhibition of bovine brain lipid peroxidation was more than 50% at 0.625 mg/mL concentration, which was among the top four of all plants studied [40].

In another study, dried aerial parts of UD were used to make a water-based extract (APUDWE) to study antioxidant activity, total phenolic content, and subsequently other biological activities (antimicrobial, antiulcer, and analgesic activities). Effective total antioxidant activity, reducing ability and superoxide radical scavenging activity were obtained by the thiocyanate method, Oyaizu method, and in the phenazine methosulfate (PMS) -nicotinamide adenine dinucleotide (NADH) systems, respectively. The total antioxidant activity of APUDWE was more than the reference compound (α-tocopherol). The chelation of ferrous ions by APUDWE was also found to be better than that of the reference compounds. The scavenging activity of APUDWE against H_2_O_2_ was found to be 23% at 250 μg [36]. The study suggested that the phenolic content may be an important contributor to the antioxidant activities of APUDWE and subsequently observed biological activities such as antimicrobial, antiulcer and analgesic activities [36,41].

In the study to calculate the antioxidant activities of medicinal plants, a thiobarbituric acid (TBA) test was conducted for an aqueous extract of UD to protect liposomes from lipid peroxidation. The inhibition of lipid peroxidation was found to be 26.7% at 500 mL/L concentration [42].

In another study, the antioxidant activity of UD was examined through cupric ion-reducing antioxidant capacity (CUPRAC), 2,2′-azino-bis(3-ethylbenzothiazoline-6-sulfonic acid (ABTS) assays, and in combination with the high performance liquid chromatography (HPLC) method along with parsley and celery leaves. UD extract (70% methanol), hydrolysate of extract, and hydrolysate of the dried plant were used for antioxidant assays. The highest antioxidant activity was observed for UD extract in the CUPRAC assay [43].

The methanolic and ethanolic root extracts of UD were studied for antioxidant activities through a DPPH assay. The methanolic root extract was found to have better antioxidant activity than the ethanolic extract in the study [44].

An extract of UD leaves was used to study antioxidant and antimicrobial activities. ferric reducing antioxidant power (FRAP), DPPH, and ABTS assays were used to study the non-enzymatic antioxidant activity of the extract, while the peroxidase activity of fresh leaves was studied as enzymatic antioxidant activity. Effective antioxidant activity was observed for the leaves of UD in all assays [45]. 

Similarly, the antioxidant activities of hydroalcoholic extracts from different parts of UD such as the flower (FUDHAE), root (RUDHAE), seeds (SUDHAE), and leaf (LUDHAE) were studied. The total antioxidant activity (TAA) was calculated through the ferric thiocyanate (FTC) method. All parts of UD had potent TAAs among them, SUDHAE had the most effective inhibition (81.7%) [17]. FUDHAE was found to have the maximum reducing power in the Oyaizu method. Superoxide radical scavenging activity at 100 μg/mL was highest in the case of SUDHAE (93.3%). Similarly, the scavenging effect of SUDHAE was highest (>60%) among the different parts of UD and standards on the DPPH method. The metal chelating (Table 1) activity was highest in the case of RUDHAE (44.2%) among the different parts of UD [17] (Table 1).

In one study, the antioxidant activity of *Taraxacum officinale* and UD leaf extracts (using ethyl acetate as solvent) were studied. The extract from UD leaves was found to have an effective antioxidant activity that was higher than the standard compound (α-Tocopherol) used in the study [46] (Table 1).

In an Iranian study, before analyzing the anticancer effect, the antioxidant activity of an aqueous extract of the leaves of UD was studied using [3-(4,5-dimethylthiazole-2-yl)-2,5-diphenyltetrazolium bromide (MTT) and FRAP methods [21]. Finally, the researcher suggested that UD could be a potential chemotherapeutic agent for breast cancer with its effective antioxidant activities [21,22].

In the same year, the antioxidant activity was studied along with the content of vitamins, minerals, and phenols of UD grown in different locations in the city of Tunceli, Turkey. In the study, effective antioxidant activity was observed, which was in the range of (IC_50_) 12.48 ± 0.466–33.70 ± 0.849 μg/mL [47]. The authors suggested the potential use of UD as an alternative natural source for the food, pharmacology and medicine sectors [47]. 

The protein fraction from the aerial parts of UD (PFAPUD) was evaluated for antimutagenic, antiproliferative and antioxidant activities. The antioxidant activity was studied through ABTS and superoxide-radical scavenger activity (SORSA) methods. The IC_50_ values for the ABTS and SORSA methods were 19.9 ± 1.0 mg/mL and 75.3 ± 0.9 mg/mL, respectively. PFAPUD was found to have better superoxide scavenging activity than the positive control (trolox) [48]. The study proposed that the radical scavenging property of the PFAPUD may also be an important factor for its antimutagenic properties [48,49].

In a comparative study, the antioxidant potential of UD leaves was studied using different extraction methods (traditional maceration extraction using ethanol and supercritical fluid extraction). The traditional ethanolic extract was found to have better antioxidant activity in the ABTS assay [50]. Similarly, in another comparative study, different extraction techniques (maceration, reflux, Soxhlet, Tillepape, and ultrasonic extraction) were used to study antioxidant activity, and phenolic and flavonoid content of leaves of UD. In all three (DPPH, FRAP, and H_2_O_2_ scavenging) assays, antioxidant activity and phenolic and total flavonoid content of the Soxhlet extraction technique were found to be the highest [51]. 

An extract of the leaves of UD rich in saponins, was studied for antioxidant activity, as antioxidant activity can help wound healing by regulating ROS [52,53]. Extract from the leaves of UD was found to have effective antioxidant activity (similar to ascorbic acid) that was important for wound healing activity of the extract [53].

In another study, before evaluating the hepatoprotective activity of the aqueous extract of UD leaves, the antioxidant activity was analyzed with the DPPH assay. The effective antioxidant activity of UD was observed in the study which inspired further hepatoprotective analysis in the mice [54].

In a further study, the different species (*Urtica dioica, Urtica urens*, and *Urtica membranacea*) of *Urtica* were analyzed for antioxidant activities through DPPH, ABTS, and FRAP assays. UD exhibited the highest antioxidant activity in all three assays which justified its medicinal importance among other *Urtica* species [55].

In a study to optimize the antioxidant activity of UD leaf extract, the two stages of leaf and extraction parameters were studied through ABTS and DPPH assays. In both assays, the highest antioxidant activities were achieved with an extract from immature leaves extracted through reflux extraction in 50% ethanol [56].

The enzymatic antioxidant activity of aqueous and ethanolic extracts of UD seeds were studied in the THP-1 cell line. Oxidative stress was caused with the help of NaF and the activity of antioxidant enzymes such as superoxide dismutase (SOD), glutathione peroxidase (GPx), and glutathione reductase (GR) were studied. Intracellular ROS were visualized during imaging which was increased in NaF treated cells and reduced with the administration of aqueous and ethanolic extracts of UD. Similarly, SOD and GPx activity were significantly increased with the addition of an ethanolic extract of UD. The study concluded that the ethanolic extract of UD seeds may have a protective antioxidant effect [37].

In a comparative analysis, the antioxidant activity of a methanolic extract of leaves of UD grown in rural (LUDR) and pollutant-exposed areas of highway (LUDP) was studied. The antioxidant activities were studied through FRAP, H_2_O_2_, and superoxide scavenging assays. Significantly higher antioxidant activities were observed in both H_2_O_2_ and superoxide scavenging assays in the case of LUDR. The study concluded that the pollutant may decrease the antioxidant potential of UD extract [57]. 

The UD leaf extraction was carried out under high pressure to increase the concertation of active compounds, and subsequently improved antioxidant activity. The antioxidant activity was calculated through the oxygen radical absorbance capacity (ORAC) assay, DNA protection assessment and DNA degradation assessment (pro-oxidant assay). The enhanced antioxidant activity was achieved in high pressure extraction conditions in all studied methods [58]. 

Similarly, a comparative study was carried out for advanced extraction techniques (microwave-assisted extraction (MAE) and pressurized liquid extraction (PLE)) with a conventional method for UD leaves (UDL) [59,60]. The objective was to achieve high concentrations of bioactive compounds such as phenolic compounds to get better biological activities including antioxidants for the UDL extract. Finally, the highest antioxidant activity of the UDL extract was observed in PLE methods, followed by MAE, and conventional methods in the ORAC assay [59].

In an interesting study, the effect of light intensity and wavelength on the antioxidant activity and content of phytochemicals were studied on micropropagated plantlets of UD. The methanolic extract of UD leaves was found to have maximum antioxidant activity at a light intensity of 130 μmol m^−2^ s^−1^. Similarly, the maximum antioxidant activity was at wavelength 1red:2.5blue [61].

In a comparative study, different drying methods were evaluated to study their impact on the phytochemicals and antioxidant activity of UD leaves. In the study, fresh, oven-dried, and heat pump-dried (HPD) leaves were used to prepare the methanolic extract. In both the DPPH and FRAP assays the extract from HPD leaves was found to have maximum antioxidant activities [62].

In a study evaluating the antiepileptic effect of UD, the antioxidant activity of UD root extract was studied in different solvents using the DPPH assay. The highest antioxidant activity was observed using ethyl acetate as a solvent, and this was selected for further antiepileptic study on a mouse model of seizure [63].

In another study, the antioxidant and other biological activities of UD, and endophytic fungi present on UD, were studied. The water and ethyl acetate extract of UD leaves were used to analyze the antioxidant activity using the DPPH assay and antioxidant enzymes (SOD and CAT) activities. The ethyl acetate extract was found to have better antioxidant activities in all tests [64].

A study was undertaken to analyze the effect of nitrogen cold plasma pre-treatment on the antioxidant activity and content of phytochemicals and other parameters of plant extracts. A total of twelve different herbs including UD were selected for the study. Plasma treatment drastically reduced the anthocyanin content of UD and low antioxidant activity was observed in the DPPH assay [39]. Recently, the evaluation of extraction techniques (ultrasound-assisted extraction with and without stirring) and solvents such as water, methanol, and ethanol was carried out. The objective of the study was to maximize the antioxidant activity of extract from the leaves of UD. Several combinations of techniques, solvents, and extraction times were used to design and conduct 36 experiments in triplicate in the study. Finally, maximum antioxidant activity using the ABTS and DPPH methods were 91.83% and 91.1%, achieved using water as a solvent in ultrasound-assisted extraction [65]. The study suggested that the aqueous extraction of UD leaves using ultrasound is an important source of natural antioxidants that can be considered as a potential substitute for synthetic additives with antioxidant activities [65]. 

In a recent study, the antioxidant activities of extract from three plants (*Urtica dioica*, *Matricaria chamomilla*, and *Murraya koenigii*) were studied through ABTS and DPPH assays. In both assays, the UD was found to have better antioxidant activity than *Matricaria chamomilla* but lower than *Murraya koenigii* [66].

In the study, the effect on antioxidant activity of UD extract by different processing (freeze-dried, oven-dried, and boiling for 5, 10, 15, and 20 min) and storage (fresh, freezer, and fridge) conditions were studied. The DPPH and ABTS methods revealed that the combination of freeze-drying and boiling leads to the highest antioxidant activity of the UD leaves [38].

In a comparative study, fresh, frozen, and dried leaves of UD were utilized to identify antioxidant activity using the DPPH and ABTS assays along with other plants. Extracts from the frozen samples had the maximum antioxidant activity in the ABTS method while in the DPPH assay the dry leaves sample was found to have maximum antioxidant activity [67].

The antioxidant potential of different leafy vegetables including UD was first studied in different solvents. UD leaf extract was found to have the highest antioxidant activity using DPPH, TAA, and reducing power potential (RPP) assays among other leafy vegetables. Therefore, UD leaves were selected for further studies of antioxidant potential in different cooking conditions (steamed and boiled) and cooking followed by simulated gastrointestinal digestion (GID). In both cases, higher antioxidant activity was observed in steam cooked and steam cooked followed by GID as compared to boiled and boiled followed by GID [68].

**Table 1:** Antioxidant activities of UD through in vitro experiments.

Methods	Extract Type/Plant Source/Dose	Reference/Standard (Activity)	Major Findings	Ref.
ABTS	MUD, MUDH, and SUDH/UD/NP	Trolox (NP)	Antioxidant activity of MUD, MUDH and SUDH were 81, 21, 9%, respectively, 66, 14, and 5%.	[43]
	Ethanolic extract/leaves of UD/20–80 μg/mL	Ascorbic acid (1.37 ± 0.01), BHA (6.58 ± 0.28) and BHT (6.27 ± 0.16)	23.55 ± 0.64 (IC_50_ (μg/gDW))	[45]
	Protein fraction/aerial parts of the UD/0.1–1200 μg/mL	Trolox (NP)	19.9 ± 1.0 (IC_50_ (mg/mL))	[48]
	Ethanolic extract/leaves of UD/5 to 40 μg/mL	Ascorbic acid (1.84 μg/mL) and BHT (6.75 μg/mL).	0.053 (IC_50_ (mg/mL))	[50]
	Hydroalcoholic extract/aerial parts of UD/NP	Trolox (NP)	2.60 ± 0.14 (TEAC)	[55]
	Ethanolic extract/immature leaves of UD/NP	(NP)	91.00 ± 1.91 (IC_50_ (μg/mL)	[56]
	Aqueous, ethanol and methanol extract/leaves of UD/2 ml	(NP)	Maximum ABTS activity (91.83%) observed for aqueous extract	[65]
	Aqueous and ethanolic extract/leaves of UD/0.5, 1.0 and 1.5 mg/mL	(NP)	ABTS: Aqueous extract 85% at 1.5 mg/mL; Ethanolic extract >80% at 0.3 mg/mL	[66]
	Aqueous extract of/fresh, frozen, and dried leaves of UD/NP	Ascorbic acid (0.0025 mg/mL)	ABTS % scavenging activity: 89.25 ± 0.04% (fresh), 90.72 ± 0.54% (frozen) and 89.17 ± 0.04% (dried)	[67]
CUPRAC	MUD, MUDH, and SUDH/UD/NP	Trolox (NP)	Antioxidant capacities MUD, MUDH and SUDH were 81, 21, 9%, respectively	[43]
DNA protection assessment	Aqueous and ethanolic extracts/leaves of UD under high pressure/NP	(NP)	148.6 ± 30.57% protection from DNA damage 35% ethanol solution at 200Mpa	[58]
DNA degradation assessment (pro-oxidant assay)	Aqueous and ethanolic extracts/leaves of UD under high pressure/NP	(NP)	In a 25% ethanol solution at 500 Mpa, DNA damage was minimum (−31.7%) with FeCl_3_	[58]
DPPH	Aqueous extract/aerial parts of UD/50, 100, and 250 μg	BHA (32%)	37% at the concentration of 60 μg	[36]
	Methanolic and ethanolic extracts/roots of UD/50, 100, 150, 200, 250, and 500 μg/mL	BHA (93.68 ± 0.0006%) and silymarin (58.14 ± 0.0005)	46.71 ± 0.0011% methanolic and 45.03 ± 0.0005% ethanolic extract at 500 μg/mL	[44]
	Ethanolic extract/leaves of UD/20–80 μg/mL	Ascorbic acid (5.36 ± 0.01), BHA (1.72 ± 0.08) and BHT (23.16 ± 0.84)	31.38 ± 0.102 (IC_50_ (μg/gDW))	[45]
	Hydroalcoholic extracts/different parts (flower, leaf, root, and seeds) of UD/50–250 μg/mL	α-tocopherol (81.4%)	Seeds (60.5%), leaf (54.2%), flower (48.7%), and root (46.2%)	[17]
	Methanol extract/aerial parts of UD/100 μg/mL	BHA (NP)	12.48 ± 0.466–33.70 ± 0.849 (IC_50_ (mg/mL))	[47]
	Methanolic extract/leaves of UD/0.05–0.3 mg/mL	BHT (0.021 ± 0.63 × 10^−3^)	0.105 ± 0.004 (EC_50_ mg/mL)	[51]
	Methanolic extract/leaves of UD/NP	Ascorbic acid (96.75%)	90.75% at 0.6 mg/mL	[53]
	Aqueous extract/leaves of UD/0.72, 1.44, 2.16, 2.88 and 3.6 mg/mL	6.13 ± 0.79 IC50 value (μg/mL)	8.73 ±0.96 IC_50_ value (μg/mL)	[54]
	Hydroalcoholic extract/aerial parts of UD/NP	Trolox (NP)	2.89 ± 0.33 (TEAC)	[55]
	Ethanolic extract/immature leaves of UD/NP	(NP)	50.39 ± 3.266 (IC_50_ in μg/mL)	[56]
	Methanolic extract/UD leaves in vitro cultured plantlets at different wavelength(1R:2.5B) and light intensity (130 μmol m^−2^ s^−1^)/NP	(NP)	IC_50_ 73.10 ± 1.31 at wavelength (1R:2.5B) 253.9 ± 6.11 at light intensity (130 μmol m^−2^ s^−1^)	[61]
	PEE, EAE, CE, EE/root of UD/50–250 μg/mL	Ascorbic acid (IC_50_ 62.08 ± 1.06 μg/mL)	(PEE, CE, EAE, and EE) in antioxidant assay were found to be 167.54 ± 1.97, 134.41 ± 0.82, 88.15 ± 1.39 and 186.38 ± 1.91 μg/mL in DPPH radical scavenging assay, respectively	[63]
	Ethyl acetate extract/leaves of UD/50–450 μg/mL	Ascorbic acid (97.30 ± 1.05%)	In DPPH assay: 94.87 ± 1.48% at 450 μg/mL	[64]
	Aqueous, ethanol and methanol extract/leaves of UD/2 mL	(NP)	DPPH (91.1%) activity observed for aqueous extract	[65]
	Aqueous and ethanolic extract/leaves of UD/0.5, 1.0 and 1.5 mg/mL	(NP)	DPPH: Aqueous extract >80% at 1.5 mg/mL; Ethanolic extract >70% at 0.3 mg/mL	[66]
	Aqueous extract of/fresh, frozen and dried leaves of UD/NP	Ascorbic acid (0.0046 mg/mL)	DPPH % scavenging activity: 15.55 ± 0.14% (fresh), 37.95 ± 0.74% (frozen) and 81.00 ± 2.33% (dried)	[67]
	Methanol, ethyl acetate, hexane extract/leaves of UD, cooked and simulated GID forms/NP	Ascorbic acid (NP)	DPPH: 18.67 μg AAE/mg, 18.47 μg AAE/mg and 0.27 mg AAE/mL for methanolic extract, steam cooked, and steam cooked and simulated GID)	[68]
FRAP	Ethanolic extract/leaves of UD/20–80 μg/mL	Ascorbic acid (143.09 ± 11.29), BHA (147.28 ± 13.87) and BHT (16.64 ± 0.30)	7.50 ± 0.43 (mM(Fe II)/g DW)	[45]
	Aqueous extract/leaves of UD/0.3–12 mg/mL	Ascorbic acid (NP)	15 g of powdered leaf of UD approximately equivalent to 30.311 mg of pure ascorbic acid.	[21]
	Methanolic extract/leaves of UD/0.05–0.3 mg/mL	Ascorbic acid 145.10 ± 10.16 (mg Fe^2+^/g dry weight)	47.71 ± 1.90 (mg Fe^2+^/g dry extract)	[51]
	Hydroalcoholic extract/aerial parts of UD/NP	Trolox (NP)	3.81 ± 0.32 (TEAC)	[55]
	Methanolic extract/leaves of UD/100 μg/mL	Trolox (NP)	313.56 ± 118.65 mg trolox/g	[57]
FTC	Hydro-alcoholic extracts/different parts (flower, leaf, root, and seeds) of UD/50–250 μg/mL	α-Tocopherol (50.1%)	Seeds (81.7%), root (79.8%), flower (78.3%), and leaf (76.4%)	[17]
	Ethyl acetate extract/leaves of UD/50 μg/mL	α-Tocopherol (65%)	76% lipid peroxidation inhibition	[46]
H_2_O_2_ scavenging activity against	Aqueous extract/aerial parts of UD/50, 100, and 250 μg	α-Tocopherol (57%)	23% at 250 μg	[36]
	Hydroalcoholic extracts/different parts (flower, leaf, root, and seeds) of UD/50–250 μg/mL	BHT (64.4%) > α-tocopherol (55.9%) > BHA (50.7%).	Root (87.3%), leaf (83.3%), seeds (74.7%), flower (71.8%)	[17]
Lipid peroxidation inhibition	Ethanolic extract/leaves of UD/0.2 and 0.625 mg/mL	Trolox (50% inhibition of lipid peroxidation)	Inhibition of bovine brain lipid peroxidation was more than 50% at 0.625 mg/mL	[40]
	Aqueous extract/aerial parts of UD/50, 100, and 250 μg	α-Tocopherol (30%)	The percentage inhibition of peroxidation in the linoleic acid emulsion was 39, 66, and 98%, at 50, 100, and 250 μg respectively,	[36]
	Aqueous extracts/UD/50, 100, 250, and 500 mg/L	Propyl gallate was used as a positive control	26.7% at 500 mg/L	[42]
Metal chelation	Aqueous extract/aerial parts of UD/50, 100, and 250 μg	α-Tocopherol (43%)	92% at 250 μg, higher than that of the reference compounds (BHA, BHT, and α-Tocopherol)	[36]
	Hydroalcoholic extracts/different parts (flower, leaf, root, and seeds) of UD/50–250 μg/mL	α-Tocopherol (53.7%)	Root (44.2%), flower (41.2%), leaf (33.6%) and seeds (31.9%)	[17]
MTT	Aqueous extract/leaves of UD/0.3–12 mg/mL	Ascorbic acid (NP)	15 g of powdered leave of approximately equivalent to 26.192 mg of pure ascorbic acid in MTT assay	[21]
Peroxidase activity	Ethanolic extract/leaves of UD/20–80 μg/mL	(NP)	1.174 ± 0.145 (μmol/mg prot/min)	[45]
ORAC	Aqueous and ethanolic extracts/leaves of UD under high pressure/NP	Trolox (NP)	266.9 ± 6.086 (mg trolox/g dry material)	[58]
	Aqueous, ethanol and acetone extracts/leaves of UD through MAE, PLE and conventional methods/NP	Trolox (NP)	ORAC (μmol Trolox equivalent/100 g) 929.80 ± 6.28 for MAE 1074.40 ± 31.20 for PLE 925.60 ± 6.70 for conventional method	[59]
	Methanolic extract/UD leaves in vitro cultured plantlets at different wavelength(1R:2.5B) and light intensity (130 μmol m^−2^ s^−1^)/NP	Trolox (NP)	(mgTE g LDW^−1^). 1356.3 ± 15.93 at wavelength(1R:2.5B) 1107.0 ± 15.92 at light intensity (130 μmol m^−2^ s^−1^)	[61]
RPP	Methanol, ethyl acetate, hexane extract/leaves of UD, cooked and simulated GID forms/NP	Ascorbic acid (NP)	9.9 μg AAE/mg for methanolic extract	[68]
Reducing capacity (Fe^3+^ to Fe^2+^)	Aqueous extract/aerial parts of UD/50, 100, and 250 μg	α-Tocopherol (NP)	Higher activities than the control (α-tocopherol)	[36]
SORSA	Protein fraction/aerial parts of the UD/0.1–1200 μg/mL	Trolox (NP)	75.3 ± 0.9 (IC_50_ (mg/mL)),	[48]
Superoxide radical scavenging activity	Aqueous extract/aerial parts of UD/50, 100, and 250 μg	α-Tocopherol (60%)	97% at 100 μg Higher superoxide radical scavenging activity than reference compounds (BHA, BHT, and α-Tocopherol)	[36]
	Hydroalcoholic extracts/different parts (flower, leaf, root, and seeds) of UD/50–250 μg/mL	BHA (67.8%) > BHT (56.4%) > α-tocopherol (44.0%).	Seeds (93.3%), root (91.1%), leaf (77.8%), flower (72.9%)	[17]
TAA	Methanol, ethyl acetate, hexane extract/leaves of UD, cooked and simulated GID forms/NP	Ascorbic acid (NP)	0.32 mg AAE/mg, 0.30 mg AAE/mg and 5.19 μg AAE/mL for methanolic extract, steam cooked, and steam cooked and simulated GID, respectively	[68]
TAC	Methanolic extract/UD leaves in vitro cultured plantlets at different wavelength(1R:2.5B) and light intensity (130 μmol m^−2^ s^−1^)/NP	Ascorbic acid (NP)	(mgAAE g LDW^−1^). 8.31 ± 0.81 at wavelength(1R:2.5B) 5.66 ± 0.40 at light intensity (130 μmol m^−2^ s^−1^)	[61]
XO inhibition	Ethanolic extract/leaves of UD/0.2 and 0.625 mg/mL	NP	>30% inhibition of XO at 0.2 mg/mL.	[40]

ABTS: 2,2′-azino-bis(3-ethylbenzothiazoline-6-sulfonic acid; BHA: butylated hydroxyanisole; BHT: hydroxytoluene; CAT: catalase; CE: Chloroform extract; CUPRAC: cupric ion-reducing antioxidant capacity; DPPH: 1,1-diphenyl-2-picrylhydrazil radical; DW: dry weight; EAE: Ethyl acetate extract; EE: Ethanolic extract; FRAP: Ferric reducing antioxidant power; FTC: Ferric thiocyanate; GID: gastrointestinal digested; MAE: microwave-assisted extraction; MTT: [3-(4,5-dimethylthiazole-2-yl)-2,5-diphenyltetrazolium bromide; MUD: hydrolysate methanolic extract of *Urtica dioica*; MUD: methanolic extract of *Urtica dioica*; NP: not provided; ORAC: oxygen radical absorbance capacity; PEE: petroleum ether extract; PLE: pressurized liquid extraction; RPP: reducing power potential; SOD: superoxide dismutase; SORSA: superoxide radical scavenger activity; SUD: solid plant hydrolysate of Urtica dioica; TAA: total antioxidant activity; TAC: total antioxidant capacity; TBA: thiobarbituric acid; TE: Trolox equivalent; TEAC: Trolox equivalent antioxidant capacity; UD: *Urtica dioica*; XO: xanthine oxidase.

### 3.2. In Vivo Antioxidant Studies

Effective antioxidant activity of UD, observed in different enzymatic and non-enzymatic assays, inspired further animal studies to utilize the antioxidant potential of UD against different diseases and conditions. The in vivo experiments conducted to analyze the antioxidant activity of UD, in blood and different organs are described in Table 2.

#### 3.2.1. Antioxidant Activity of UD Studied in Brain

A study was carried out to analyze the effect of UD dried leaf supplementation (UDLS) and exercise on oxidant stress in the brain of healthy Wistar rats. UDLS significantly reduced the free electron accumulation, in both the frontal lobe and cerebellum of the brain compared to both the exercise and control groups [69]. UDLS was found to be an effective antioxidant that may be helpful in recovery from brain injury. It could be developed as an antiapoptotic supplement, promoting cell survival in the brain [69,70].

In a similar study, the protective effects of UD on the striatal mito-oxidative damage of 1-methyl-4-phenyl-1,2,3,6-tetrahydropyridine (MPTP)-induced behavioral, cellular, and neurochemical alterations in rats were studied. The ethyl acetate fraction of the extract significantly decreased MDA and nitrite concentration, in both the striatum and cortex regions of the brain in the rats, which were elevated due to the MPTP treatment. Decreased activity of GSH and catalase in the striatum and cortex regions of the brain of MPTP-treated rats was also restored with the application of UD [71]. In the study, the ethyl acetate fraction of the UD extract showed neuroprotective actions through antioxidant and anti-inflammatory activities in the brain that supported the rationale for clinical use of UD in the treatment of movement disorders [71,72].

An extract from the aerial parts of UD (EAUD) was used in the study to protect neuronal oxidative damage in scopolamine induced memory impairment in rats. The three concentrations of EAUD (20, 50, or 100 mg/kg) used in the study were effective in reducing the effect of scopolamine by decreasing the concentrations of malondialdehyde (MDA) and improving the level of thiol in the hippocampus and cortex regions of the brain. CAT activity was also significantly increased with all three concentrations of EAUD in the hippocampus region. However, in the cortex region, the CAT activity increased significantly only with two concentrations (50 or 100 mg/kg) of EAUD. Superoxide dismutase (SOD) activity was also found to increase significantly with two concentrations (50 or 100 mg/kg) of EAUD in the cortex and hippocampus regions of the brain [73]. Similar to a previous study, it also strongly supported the neuroprotective properties of UD extract via antioxidant activities [73,74]. In a recent study, the root extract of UD was also studied along with the leaf extract for antioxidant properties against scopolamine-induced neuropathies in rats. Both UD extracts have anti-oxidative effect in the brain as MDA, nitric oxide, oxidized glutathione (GSSG) and oxidized GSSG percentage levels were found to be significantly decreased in the brain, which were significantly increased due to scopolamine administration in rats. Further, the concentration of reduced GSH and the ratio of GSH/GSSG were found to be significantly increased, which were reduced due to scopolamine treatment [75]. The authors suggested that the antioxidant effect of UD, especially UDR, may counteract neurodegenerative diseases such as Alzheimer’s disease [75,76].

In another recent study, the antioxidant properties of UD extract in a mouse model of multiple sclerosis were studied. The MDA level in brain tissues was found to be significantly reduced in all UD treatment groups significantly, in a dose–dependent manner as compared to the positive control group. TAC was also found to increase in all treatment groups, but it was only significant in the second highest dose group (200 mg/kg of UD extract). It was also highest in comparison with all other groups, including both positive and negative control groups. The study suggested that the potent antioxidant activity of UD extract is useful in neuroprotection, which might be effective to treat and mitigate demyelination [77,78]. 

Recently, the antioxidant effect of the aerial parts of UD was studied in the hippocampus region of healthy rats alone (only UD treatment (UDT)) and in combination with resistance exercise (UDT+RE). The study was designed to examine the role of UDT and RE in controlling premature aging and memory impairment. Antioxidant activities were observed in both cases (UDT and UDT+RE). A decrease in the MDA level was observed in both the UDT and UDT+RE groups. However, it was only significant later. Similarly, a significant increase in GSH and Glutathione peroxidase (GPx) was also observed in both the cases of UDT and UDT+RE [79]. Overall, the study suggests that the regular UDT+RE may reverse the decrease in some aging related enzymes in the hippocampus region and enhance cognition [79].

#### 3.2.2. Antioxidant Activity of UD Studied in Liver

The antioxidant effect of UD on liver regeneration was studied in rats after partial hepatectomy. UD treatment significantly decreased the elevated tissue MDA level and increased the reduced SOD activity and GSH level in the liver tissues [80]. Pretreatment with UD was suggested to be helpful in liver protection and liver regeneration after partial hepatectomy [80,81]. 

In a different study, the hepatoprotective effect of a hydroalcoholic extract of UD (whole plant) based on its antioxidant properties, was studied in rats challenged with CCl_4_. The potent antioxidant fraction (ethyl acetate) of the extract significantly decreased the elevated level of MDA and nitrite concentration. Similarly, the treated group of rats significantly restored the level of endogenous antioxidant enzymes such as catalase and reduced GSH, in a dose–dependent manner [82]. Like previous studies antioxidant properties of UD were strongly suggested for hepatoprotective effect in rats and as a potential therapeutic for liver disorders.

#### 3.2.3. Antioxidant Activity of UD Studied in Blood

In an in vivo study, the antioxidant effect of UD seed oil was studied alone and in combination with the seed oil of *Nigella sativa*. Treatment with UD seed oil alone decreased the elevated level of MDA in the blood, and increased antioxidants such as glutathione (GSH), ceruloplasmin, vitamin E, vitamin C, beta-carotene and retinol in the serum of CCl_4_-treated rats [83]. It was suggested that the properties of UD observed in the study, such as the increase in antioxidants and the decrease in lipid peroxidation may be utilized in hepatoprotective therapies [83,84,85].

In another study, broiler chickens were used to study the antioxidant and immunomodulatory effects of UD powder as a supplement. The antioxidant activity of blood serum was found to be higher in the TAC assay in comparison with the control group after 42 days of treatment [86].

A leaf extract of UD was studied against oxidative stress and blood pressure modulation in male spontaneously hypertensive rats (SHR). In the study, a significant increase in the activity of SOD and CAT in erythrocytes was observed at all doses tested. Similarly, plasma lipid peroxidation of SHR was reduced in the thiobarbituric acid reactive substances (TBARS) assay. Furthermore, the ferric reducing antioxidant power of plasma (FRAPp) and the antioxidant capacity of plasma (TEACp) were found to increase significantly in all treatment groups. The authors concluded that the antioxidant property of UD may be an important contributor to its antihypertensive effect [87].

A dietary supplement of hydroalcoholic extract of UD was provided to study its effect on antioxidant status, body growth, and other biochemical parameters in broiler chickens. Three different concentrations were used in the study to optimize its effect. Finally, the 0.5 mg/L dose of UD extract was selected where the highest antioxidant activity (decrease in MDA level and increased TAC, GPx, and SOD) was achieved in the blood samples [88].

#### 3.2.4. Antioxidant Activity of UD Studied on Kidney

The antioxidant effect of the methanolic extract of the leaves of UD was studied against acute kidney injury in rats. The production of ROS (such as hydroxyl radicals and superoxide anion) in the kidney is an important characteristic of gentamycin-induced injury [89]. An elevated MDA level was observed in kidney tissue that indicated increased lipid peroxidation and the involvement of free radicals in the gentamycin treatment group. The application of UD significantly reduced the elevated level of MDA in the group that received both gentamycin and UDLE (GUDLE). Similarly, FRAP was significantly increased in the GUDLE group, which was significantly reduced due to gentamycin induced injury. The antioxidant activity of the extract was suggested to be the main factor behind the nephroprotective effect of UDLE [90,91].

#### 3.2.5. Antioxidant Activity of UD Studied in Muscle Tissue

Muscle ischemia reperfusion is frequent in humans, and can lead to serious consequences in some cases [92]. ROS have an important role, which was apparent in several studies that generally showed a protective effect of antioxidant therapies during ischemia reperfusion of the muscles [93]. The antioxidant effect of leaf extract from UD was studied in the muscles of rats. Oxidative stress caused by the application of a tourniquet in the muscles of rats was studied by analyzing the MDA level. The MDA level of muscle samples from the rats treated with UD was lower than those of KCl treated and untreated groups after both 1 or 2 h of ischemia [94]. Pre-treatment with an extract from UD may counter the oxidative stress in the muscles generated by applying a tourniquet. Therefore, more studies are proposed to develop it for patients undergoing pneumatic tourniquet operations to reduce oxidative damage in skeletal muscles [94].

#### 3.2.6. Antioxidant Activity of UD Studied in Heart Tissue

The cardioprotective effect of UD was studied in rats treated with doxorubicin. Doxorubicin causes oxidative stress which may be the major factor behind its cardiotoxicity. Treatment with seed oil of UD significantly decreases the MDA level, and increases the activity of antioxidant enzymes such as SOD, CAT, and GSH-Px [95].

#### 3.2.7. Antioxidant Activity of UD Studied in Different Organs

In a primary study, the extract of UD leaves was analyzed for its effects on different enzymes including antioxidant enzymes, in the liver, kidney, forestomach, and lungs of mice. Activities of important antioxidant enzymes such as SOD and catalase (CAT) were found to be significantly increased in all organs analyzed in the study as compared with the control (Table 2) [96]. The study concluded that the significant antioxidant activity of UD leaves can be utilized in chemotherapy against multiple organs (Figure 1) [96,97,98].

In another study, the hepatoprotective and antioxidant properties of UD seed extract were studied in various tissues of rats. Aflatoxin was used to cause oxidative stress and hepatic-toxicity in the rats. The MDA level, which was increased significantly in the aflatoxin administered group, reduced in the UD treatment group in different tissues from liver, kidney and erythrocytes. Similarly, UDS administration significantly increased the levels of antioxidants, particularly CAT, GR and SOD, in the liver. The hepatoprotective effect of UD treatment in the study may be attributed to the antioxidant properties of UD [99,100].

Antioxidant effects of a UD aqueous extract on asthmatic pathology, was also studied in a rat asthma model. UD treatment in rats exposed to ovalbumin (OA)-induced inflammation, significantly reduced MDA levels in the lungs compared to OA-sensitized rats. Although not significant, reduction of MDA levels was also observed in liver and erythrocytes [101]. The study confirmed that the administration of UD extract could be responsible for the protective effects against airway inflammation through antioxidant activities [101,102].

ROS generation is shown to be associated with the development of pulmonary hypertension syndrome (PHS) in broiler chickens. Hence, the antioxidant activity of UD was studied in broiler chickens (Ross 308 strain). Three concentrations (0.5, 1 and 1.5%) of dried leaf and stem of UD were added to chicken feed for 42 days. In serum samples, MDA concentration was significantly reduced in the treatment group and nitric oxide was increased. SOD1 and CAT were found to be significantly overexpressed in both liver and lungs of treatment groups, having a 1% or 1.5% dose of UD, which may be a reason for its antioxidant effects. The same concentrations of UD also significantly reduced mortality up to 42 days of age in broiler chickens [103]. The antioxidant activity of UD was suggested to be an important factor for these positive outcomes in the study, since feeding with UD at different concentrations attenuated right ventricular hypertrophy in the study, which was also associated with a significantly reduced mortality rate [103].

The effect of UD leaf extract was also studied against oxidative status in polycystic ovary syndrome in a mouse model, alone and in combination with lutein. A reduction in MDA level (in the ovary, uterus and serum) was observed in the high UD leaf extract treatment group and the maximum reduction was observed in the group that received both UD leaf extract and lutein. Similarly, total antioxidant capacity (TAC) was found to be increased in the high UD leaf extract treatment group, and the maximum increase in the treatment groups was seen in the group that received both UD leaf extract and lutein. Based on these results, the study concluded that oral administration of the UD extract, alone and in combination, improved reproductive function by improving antioxidant activity [104].

Antioxidant activity and protective effects of UD seed extract were analyzed in irradiated rats. MDA levels and antioxidant enzymes such as SOD, CAT, GSH, and GSH-Px were studied in the serum and liver of the rats. SOD was found to be highest in serum and liver of the UD treatment group. Similarly, GSH-Px activity was found to be highest in the serum of the UD treatment group and lowest in the irradiated group. The GSH-Px activity was found to be higher in the liver compared to the irradiated group. More studies with more animals are suggested to achieve significant results for other antioxidant enzymes [105]. 

**Table 2:** Antioxidant activities of UD through in vivo experiments.

Animal Model Oxidative Stress	Material, Dose and Duration	Method	Major Findings	Ref.
Wistar rats provided Swimming training	Dried leaf of UD 1% *w*/*w* for 8 weeks	The level of oxidative stress was measured by EPR	Reduction in the free electron accumulation, both in the frontal lobe or in the cerebellum compared to the control and exercise groups	[69]
Male Wistar rats MPTP induced oxidant stress (model of Parkinsonism)	Hydroalcoholic extract for UD (whole plant) in EAF (20, 40, and 80 mg/kg, p.o.) for 14 days	Measurement of lipid per oxidation, nitrite, reduced glutathione levels, and catalase estimation were carried out in liver	MDA level↓and nitrite↓, CAT↑ and reduced GSH↑ in dose dependent manner	[71]
Male Wistar rats scopolamine-induced memory impairment model	Hydroalcoholic extract from the aerial parts of UD (20, 50, or 100 mg/kg IP for two weeks	MDA, thiol, SOD, and CAT were studied in the hippocampus and cortex region of brain	In the hippocampus: MDA↓, thiol↑, CAT↑ and SOD↑ In cortex: MDA↓, thiol↑, CAT↑ and SOD↑	[73]
Male, 4-month-old albino rats Scopolamine-induced neuropathies in rats	Leaves and root extracts from UD extract (100 mg/kg) by oral gavage daily for 19 days	MDA, NO, reduced GSH and oxidized GSSG levels of glutathione in brain tissues was determined by HPLC	In the brain: MDA↓, NO↓, GSSG↓, Oxidized GSSG percentage↓, GSH↑, and ratio of GSH/GSSG↑	[75]
Male C57BL/6 mice Cuprizone-induced demyelination or accelerate remyelination in a mice model	Hydroalcoholic extract from the aerial parts of UD by oral gavage 50, 100, 200, and 400 mg/kg for 21 days (three times a week)	MDA and TAC of brain tissues were measured	In brain tissues: MDA↓ and TAC↑	[77]
Male Wistar rats resistance training	Hydroalcoholic extract of aerial parts of UD. A dose of 50 mg/kg in 0.0166 *w/v* (500 mg of UD extract per 30 mL of distilled water) daily through oral gavage	Hippocampus was used to measure the levels of MDA, SOD, CAT activity, GPx, and GSH enzymes	In the hippocampus: GPx↑,GSH↑, and MDA↓	[79]
Male Sprague-Dawley rats Stress induced through partial hepatectomy in rats	Oil from the UD. 2 mL/kg/day) once a day orally for 7 days starting 3 days prior to hepatectomy operation	MDA level and enzymatic activities of SOD activity and GSH in the hepatic tissues	In liver tissue: MDA level↓, SOD and GSH↑ as compared to PH group	[80]
Young Wistar rats CCl_4_ induced oxidant stress and hepatotoxicity	Hydroalcoholic extract for UD (whole plant) in ethyl acetate fraction. EAF and a dose of 20, 40, and 80 mg/kg BW for 7 days	Measurement of lipid per oxidation, nitrite, reduced glutathione levels, and catalase estimation were carried out in liver	MDA↓, nitrite↓, catalase↑ and reduced GSH↑	[82]
Male Sprague–Dawley rats Oxidative stress caused by CCl_4_ treatment	Oil from the seed of UD through IP of 2 mL/kg for 45 days	Blood MDA level (nmol/mL of erythrocytes) and serum antioxidant levels (mg/dl) for GSH, ceruloplasmin, vitamin E, vitamin C, beta-carotene, and retinol were measured	MDA level↓, GSH↑, ceruloplasmin↑, vitamin E↑, vitamin C↑, beta-carotene↑ and retinol↑	[83]
Wistar albino rats Oxidative stress caused by CCl_4_	Oil from the seed of UD, IP of 2 mL/kg of UD oils for 60 days treatment	Blood MDA level (nmol/mL erythrocytes) and serum antioxidant levels (mg/dL). GSH, ceruloplasmin, vitamin E, vitamin C, beta-carotene, and retinol	MDA level↓, GSH↑, ceruloplasmin↑, vitamin E↑, vitamin C↑, beta-carotene↑ and retinol↑	[85]
Broiler chickens (Ross 308 strain)	2 g/kg UD powder for 42 days	TAC of blood serum	1.142 TAC↑ compared to control 1.286 (mmol/dL) vitamin E	[86]
Male, spontaneously hypertensive rats Losartan induced hypertension	Methanol extract of UD leaves with ultrasound-assisted extraction technique 10, 50, and 200 mg/kg/day of UE for 4 weeks	SOD, CAT, ferric reducing antioxidant power of plasma (FRAPp) and antioxidant capacity plasma (TEACp) from the blood was studied	CAT↑, SOD↑, FRAPp↑ and TEACp↑	[87]
Male broiler chickens (Ross 308)	Hydroalcoholic extract of UD at 0.25, 0.5, and 1 mL/L for 42 days	(TAC), total SOD, and GPx in plasma were assayed using colorimetric methods	In comparison with control at 0.25 and 0.5 mL/L: MDA↓ and TAC↑ GPx↑ SOD↑	[88]
Albino Wistar rats Gentamicin-induced kidney injury induced by gentamicin in rats	Methanolic extract of UD leaves, (200 mg/kg/day, gavage) for 8 days	FRAP and lipid peroxidation through MDA in renal tissue	In renal tissue: MDA↓ and FRAP↑	[90]
Male Wistar rats Oxidative stress caused through tourniquet application	Leaf extract of UD 1.15% KCl solution provided 500 mg/100 g BW in 2.5 mL of KCl aqueous solution once a day through intra esophageal cannula for 5 days	500 mg/100 g body weight of the UD extract in 2.5 mL of aqueous solution of KCl	MDA level of muscle sample of UD treated group was lower than those of KCl treated and untreated groups after both 1 or 2 h ischemia	[94]
Eighteen female Sprague-Dawley rats Oxidative stress provided through doxorubicin	Seed oil of UD at 2 mL/kg for 14 days	MDA (nmol/mg protein), SOD (U/mg protein), CAT (U/mg protein), and GSH-Px (U/g protein) were measured in heart tissues	In comparison with doxorubicin group: MDA↓, GSH_Px↑, CAT↑ and SOD↑	[95]
Swiss albino male mice No stress provided	Hydroalcoholic extract of leaves from UD, 50–100 mg/kg body weight (BW) oral gavage daily for 14 days	Determination of LPO and enzymatic assay was used for CAT, SOD, GPx, GST, LDH, and GR	In the liver SOD↑, CAT↑, GPx↑, GR↑, LPO↑, LDH↓ and GST↑ In forestomach SOD↑, CAT↑ and GST↑ In kidney SOD↑, CAT↑ and GST↓ In lungs SOD↑, CAT↑ and GST↓	[96]
Male Sprague–Dawley rats Received aflatoxin-induced tissue injury	UD seed extract in diethyl ether as solvent, 2 mL of UDS oils/rat/day by gavage for 90 days	antioxidant capacity measured through serum marker, enzymes, antioxidant defense systems, and lipid peroxidation through level MDA	In the liver: MDA level↓, CAT↑, SOD↑, and GR↑ In erythrocytes: MDA level↓ In kidney: MDA level↓	[99]
Male Wistar rats Ovalbumin-induced inflammation	Aqueous extract from the leaves of UD (1.5 g/kg/day) orally for 25 days	MDA, GPx, GSH, SOD and CAT were studied in erythrocytes liver and lungs	MDA levels↓ in the lungs	[101]
Broiler chickens (Ross 308 strain) Birds were reared for 6 weeks in a high-altitude region (2100 m)	The dried leaves and stem of UD (0.5, 1 and 1.5%) were added to chicken feed for 42 days	NO and MDA concentrations were measured in serum samples. Expression of SOD1 and CAT was studied in both liver and lungs	In serum: NO↑, MDA↓ In lungs and liver: SOD1↑ and CAT↑	[103]
NMRI mice Induction of polycystic ovary syndrome (PCOS) by dehydroepiandrosterone	Hydroalcoholic extract from the aerial parts of UD at 200 and 400 mg/kg for 30 consecutive days after PCOS induction	The level of MDA and TAC were studied in serum ovary and uterus samples	In serum, ovary, and uterus: MDA↓ and TAC↑	[104]
Male Wistar albino rats Oxidative stress provided through radiation	Ethanolic seed extract of UD at 30 mL/kg for 10 days	MDA, CAT, SOD, GSH, and GSH-Px	In comparison with irradiated group in serum: SOD↑, GSH↑, and GSH-Px↑ In liver: GSH-Px↑	[105]

BW: body weight; CAT: catalase; EPR: electron spin resonance; FRAPp: reducing antioxidant power of plasma; GPx: glutathione peroxidase: GSH: glutathione; GST: glutathione S-transferase; GR: glutathione reductase; GSSG: oxidized glutathione; HPLC: high performance liquid chromatography; IP: intraperitoneal injection; LDH: lactate dehydrogenase; LPO: lipid peroxidation; MDA: malondialdehyde; MPTP: 1-methyl-4-phenyl-1,2,3,6-tetrahydropyridine; NO: Nitric oxide; PCOS: polycystic ovarian syndrome; PH: partial hepatectomy; SHR: spontaneous hypertensive rat; SOD: superoxide dismutase; TAA: total antioxidant activity; TAC: total antioxidant capacity; TEACp Trolox equivalent antioxidant capacity of plasma; UD: *Urtica dioica*; ↑: increase; ↓: decrease.

### 3.3. Antioxidant Activity of UD Studied in Humans

A large number of clinical trials and human studies have been conducted against several diseases and conditions using UD [106,107,108,109,110,111]. These studies mostly evaluated the anti-diabetes anti-cancer and anti-inflammatory potential in patients [106,107,108,109,111]. However, the antioxidant potential of UD has only been analyzed in a few human studies (Table 3). 

In a randomized double blind clinical trial, the antioxidant effect of a hydroalcoholic extract of aerial parts of UD was studied in patients suffering from type 2 diabetes. The effect of the extract against oxidative stress was analyzed in the patients through a randomized double blind clinical trial composed of 27 men and 23 women (Table 3). After the 8 weeks of treatment, compared to control group, the antioxidative markers from blood such as TAC and SOD showed a significant increase in the treatment group [107]. Results supported the concept that UD administration can protect the body against free radicals and diabetes complications. However, no significant differences were observed in MDA levels and glutathione oxidase in the study. The authors suggested that the antioxidant properties of UD may help to protect against cardio vascular diseases in the patients suffering from type-2 diabetes [107,112]. 

In a different double blind, randomized clinical trial, the UD extract was used to study antioxidant effects on 50 women with type 2 diabetes. Biochemical analysis of blood samples for diabetes and antioxidant-related parameters before and after 8 weeks of treatment, included SOD and NO. Both SOD and NO were found to increase significantly in the treatment group. In line with the previous clinical trial study, the antioxidant activity of the extract was thought to be an important factor for the improvement of diabetes-related parameters in the participants in the treatment group [106,107].

In a study on patients with inflammatory bowel disease (IBD), the hydroalcoholic extract of UD leaves was used as a tablet in a double-blind, placebo-controlled, randomized, clinical trial. After 12 weeks of treatment, the blood level of the antioxidant enzyme SOD, was found to be significantly increased in the treatment group. Oxidative stress is also proposed as an important mechanism underlying the pathophysiology of IBD. The antioxidant activity of UD in the treatment group may be an important factor in the decrease in inflammation and increase in the quality of life of the patients [113]. 

In a recent double-blind randomized clinical trial, 60 men suffering from benign prostatic hyperplasia (BPH) were treated with UD root extract or placebo three times a day for 12 weeks. Antioxidant activity was measured from the blood samples that included GSH, MDA and SOD activities through a colorimetric method with a spectrophotometer. In the study, a significant decrease in the level of MDA was observed, suggesting reduced lipid peroxidation in the UD treatment group. Similarly, a significant increase in SOD activity was also observed in the study, as in previous clinical trials (Table 3) [106,107,108]. Overall, UD administration results in an improvement in international prostate symptoms score (IPSS), an increase in antioxidants, and a decrease in inflammatory biomarkers. In general, the study demonstrated the effectiveness of the UD extract against BPH and enhanced antioxidant activities [108].

**Table 3:** The antioxidant activity of UD studied in humans.

Clinical Trial	Material, Dose and Duration	Method	Major Findings	Ref.
Randomized double blind clinical trial composed of 27 men and 23 women suffering from type 2 diabetes	Hydroalcoholic extract of aerial parts of UD. Dose of 100 mg/Kg for 8 weeks	TAC, SOD, MDA and glutathione oxidase activity were studied in the blood of the participants	In blood: TAC↑ and SOD↑	[107]
Randomized double blind clinical trial composed of 50 women with type 2 diabetes	Hydroalcoholic extract from the aerial parts of UD. 5 mL of extract or placebo in three portions a day (every 8 hr), for 8 weeks	SOD and NO in the blood of the participants	In blood: NO↑ and SOD↑	[106]
Randomized double blind clinical trial composed of 64 patients with inflammatory bowel disease	Hydroalcoholic extract of UD leaves, (400 mg) three times for 12 weeks	SOD in blood of the participants	In blood: SOD↑	[113]
Randomized double-blind clinical trial consisting of 60 men with BPH	Root extract of UD capsules (150 mg) three times per day for 12 weeks	Blood samples of the participants were used to study GSH, MDA and SOD activity	In blood: MDA↓ and SOD↑	[108]

BPH: benign prostatic hyperplasia; CAT: catalase; GSH: glutathione; MDA: malondialdehyde; NO: Nitric oxide; SOD: superoxide dismutase; TAC: total antioxidant capacity; UD: *Urtica dioica*; ↑: increase; ↓: decrease

## 4. Conclusions and Future Suggestions

In initial studies, the antioxidant potential of UD was discovered in the screening of edible and medicinal plants in different studies as UD falls into both categories [40,42]. These initial studies highlighted the antioxidant activities of UD through standard assays that paved the path to exploiting its antioxidant potential. Further, different methods (non-enzymatic and enzymatic) have been used to study the antioxidant activities of the UD in detail. In these studies, different parts of the UD were used, such as leaves, roots, flower seeds, and aerial parts [17,75,83]. In some comparative analysis, the antioxidant potential of flowers, seeds, and roots were higher than that of leaves [17,75]. However, leaves are preferred in most studies, as these are commonly used as food [17]. Relatively less utilized parts of the UD such as flowers may also be explored for antioxidant activities against different diseases, as in some studies these parts had higher antioxidant activity than the leaves [17]. In recent studies, researchers have optimized extraction parameters and used advanced techniques such as microwave-assisted, ultrasound-assisted and pressurized liquid extractions to achieve higher content of bioactive compounds and subsequently antioxidant activity of UD [58,59,65]. There is a need to explore these extracts (optimized through advanced extraction techniques) with high antioxidant activities in future research for pharmacological experiments as therapeutic candidates. Limited studies have been conducted to identify phytocompounds responsible for antioxidant activity in the extract of UD, as phenolic compounds known in the extract are considered responsible for antioxidant activity [36]. Some studies have indicated the role of nonphenolic compounds in the antioxidant activity of UD, as in the comparative studies the fraction of extract from UD with more non-phenolic compounds was reported to have more antioxidant activity [59]. Precise knowledge of the phytochemical(s) responsible for antioxidant activity may be helpful to optimize and harvest the maximum potential of antioxidant activity of the plant. It is strongly suggested in future studies to discover the individual phytochemicals present in the UD responsible for antioxidant activity. Similarly, limited studies have been conducted to identify the molecular mechanism and signaling pathway targeted through the antioxidant activity of UD in different diseases such as cancer and diabetes. The knowledge of targets and associated pathways of the antioxidants present in UD may be highly useful in developing UD as a therapeutic candidate against these important diseases.

Few studies have been conducted to explore the antioxidant potential of UD in humans despite the support of several in vitro and in vivo studies, as well as a strong safety profile from its historical use as a food. In human studies, the antioxidant activity of UD has been considered only in three diseases (i.e., diabetes, IBD and BPH) to date [106,107,108,111]. More human studies are required to establish the antioxidant potential of UD as a therapeutic against these diseases. The antioxidant activity and application of UD were found to be effective in other important diseases and different organs (Figure 1) [19,33]. As a result, after considering the associated safety issues, it is suggested the antioxidant effect of UD be studied in patients with other diseases, such as various cancers and neurological disorders. It should be noted that the antioxidant activity of UD has a huge potential for different biological activities that can be boosted through filling current gaps and following the suggested direction of action in the current study.
